# Predicting response to vascular endothelial growth factor inhibitor and chemotherapy in metastatic colorectal cancer

**DOI:** 10.1186/1471-2407-14-887

**Published:** 2014-11-27

**Authors:** Petra Martin, Sinead Noonan, Michael P Mullen, Caitriona Scaife, Miriam Tosetto, Blathnaid Nolan, Kieran Wynne, John Hyland, Kieran Sheahan, Giuliano Elia, Diarmuid O’Donoghue, David Fennelly, Jacintha O’Sullivan

**Affiliations:** The Centre for Colorectal Disease, St. Vincent’s University Hospital, Dublin 4, Ireland; College of Life Sciences, University College Dublin, Dublin 4, Ireland; Conway Institute, University College Dublin, Dublin 4, Ireland; Department of Surgery, Trinity Centre for Health Sciences, Institute of Molecular Medicine, St. James’s Hospital, Dublin 8, Ireland

**Keywords:** Colorectal cancer, Bevacizumab, 2D-DIGE, Biomarker, Proteomics

## Abstract

**Background:**

Bevacizumab improves progression free survival (PFS) and overall survival (OS) in metastatic colorectal cancer patients however currently there are no biomarkers that predict response to this treatment. The aim of this study was to assess if differential protein expression can differentiate patients who respond to chemotherapy and bevacizumab, and to assess if select proteins correlate with patient survival.

**Methods:**

Pre-treatment serum from patients with metastatic colorectal cancer (mCRC) treated with chemotherapy and bevacizumab were divided into responders and nonresponders based on their progression free survival (PFS). Serum samples underwent immunoaffinity depletion and protein expression was analysed using two-dimensional difference gel electrophoresis (2D-DIGE), followed by LC-MS/MS for protein identification. Validation on selected proteins was performed on serum and tissue samples from a larger cohort of patients using ELISA and immunohistochemistry, respectively (n = 68 and n = 95, respectively).

**Results:**

68 proteins were identified following LC-MS/MS analysis to be differentially expressed between the groups. Three proteins (apolipoprotein E (APOE), angiotensinogen (AGT) and vitamin D binding protein (DBP)) were selected for validation studies. Increasing APOE expression in the stroma was associated with shorter progression free survival (PFS) (p = 0.0001) and overall survival (OS) (p = 0.01), DBP expression (stroma) was associated with shorter OS (p = 0.037). Increasing APOE expression in the epithelium was associated with a longer PFS and OS, and AGT epithelial expression was associated with a longer PFS (all p < .05). Increasing serum AGT concentration was associated with shorter OS (p = 0.009).

**Conclusions:**

APOE, DBP and AGT identified were associated with survival outcomes in mCRC patients treated with chemotherapy and bevacizumab.

**Electronic supplementary material:**

The online version of this article (doi:10.1186/1471-2407-14-887) contains supplementary material, which is available to authorized users.

## Background

Colorectal cancer is the second leading cause of death from cancer in the western world [1]. Up to 50% of patients at presentation have metastatic disease [2]. Survival has increased in the past decade to approximately two years in these patients with the introduction of irinotecan and oxaliplatin chemotherapy, as well as the use of targeted therapies such as cetuximab (Erbitux) that targets the EGF receptor, and bevacizumab (Avastin), a humanized monoclonal antibody to vascular endothelial growth factor-A (VEGF-A) [3]. However, response rates of less than 50% have been reported with these drugs [4, 5]. *KRAS* mutations are a predictor of resistance to anti-EGFR monoclonal antibodies in CRC, however clinical benefit from anti-VEGF therapy is independent of *KRAS* status [6, 7]. Biomarkers predictive of bevacizumab response are lacking not only in mCRC, but in all diseases in which bevacizumab is used. Biomarkers are urgently required to improve cost effective treatment and avoid unnecessary toxicity for patients who are unlikely to respond.

Many studies on the identification of predictive biomarkers to bevacizumab have been performed. Much focus has been on VEGF-A, a proangiogenic ligand which is selectively inhibited by bevacizumab. One study assessed the prognostic and predictive use of circulating VEGF-A levels in phase III trials of bevacizumab involving 1,816 patients with colorectal, lung and renal cell carcinoma [8]. Plasma pretreatment VEGF-A levels were prognostic for outcome in mCRC, lung and renal cell cancers, but were not predictive for bevacizumab benefit. However, VEGF concentrations are dynamic, and therefore pretreatment levels may not reflect treatment related changes [7]. Keskin et al. assessed serum VEGF and basic fibroblast growth factor (bFGF) in mCRC patients treated with FOLFIRI and bevacizumab [9]. Pre and post-treatment serum levels were decisive in evaluating response to treatment and prognosis. Serum VEGF and bFGF levels were significantly higher than the healthy controls, and patients with high pre-treatment serum bFGF levels had significantly shorter PFS. In addition,VEGF-A expression in IHC and *in situ* hybridisation was not a predictive marker for bevacizumab efficacy in mCRC patients [10].

Proteomic techniques have been used to investigate the mechanisms of resistance to targeted therapies and chemotherapy, as well as identify biomarkers which may predict response, including biomarkers to bevacizumab. One study assessed the predictive and/or prognostic serum proteomic biomarkers in patients with epithelial ovarian cancer (EOC) as part of the ICON7 clinical trial [11]. The ICON7 trial was a phase III trial in patients with EOC who were randomized to carboplatin/paclitaxel chemotherapy or to this regimen plus bevacizumab. PFS was statistically better in the bevacizumab arm, however absolute benefit was only 1.5 months. Serum samples from ten patients who received bevacizumab were divided into responders and non-responders. Serum samples were depleted of the fourteen most abundant proteins, and samples were then analysed by mass spectrometry (MS) to identify candidate biomarkers. Three candidate biomarkers were identified. When these markers were combined with CA125, a discriminatory signature identified patients with EOC who were more likely to respond to bevacizumab. Validation in further patient cohorts is required.

Although proteomics has been used in the investigation of targeted therapies in cancer, and many potential biomarkers have been identified in the discovery phase, few biomarkers have undergone validation. The identification of biomarkers that will allow for the prediction of patients who respond to a particular treatment, has the potential to individualize treatment, thereby maximizing benefit and avoiding unnecessary expenditure and toxicity in those unlikely to respond.

**Figure 1:**
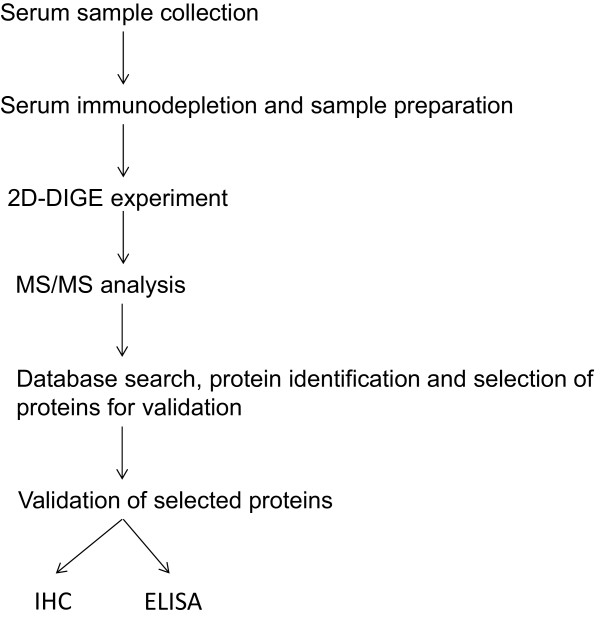
**Experimental workflow.**

In this study, we explored the hypothesis that a patient’s lack of response to bevacizumab is a result of differentially expressed proteins. We used a 2D- differential gel electrophoresis (2D-DIGE) approach to investigate the serum of patients with mCRC in order to determine if differential protein expression can differentiate responders to bevacizumab and validated select proteins with ELISA and IHC (Figure 1).

## Methods

### Treatment groups and sample collection

The acquisition of patients’ serum and paraffin tissue specimens was approved by the ethics committee at St. Vincent’s University Hospital, Dublin, Ireland. Blood samples were collected from patients diagnosed with mCRC prior to commencing chemotherapy and bevacizumab (Genentech; 5-7.5 mg/m^2^ every 2-3 weeks). Informed consent for participation in the study was obtained from participants. Paraffin tissue specimens were collected following surgical resection and prior to receiving chemotherapy and bevacizumab. Blood samples were collected in anticoagulant free tubes, allowed to coagulate at room temperature for 15 min and then centrifuged at 2000 rpm for 10 min at 20°C. Serum was then aliquoted and stored at -80°C until time of analysis. An initial biomarker discovery cohort of patients were divided into responders (n = 11) and nonresponders (n = 12). Patients were divided according to the PFS, time from diagnosis of metastatic disease until radiological progression which resulted in change of treatment while on bevacizumab. Patients with greater than nine months (270 days) PFS were classified as responders. This timeframe was chosen based on the N016966 phase III trial assessing the efficacy of bevacizumab with either capecitabine and oxaliplatin (XELOX) or FOLFOX-4 in the first- line setting of patients with mCRC [12]. PFS was significantly increased in the bevacizumab arm compared with placebo when combined with oxaliplatin-based chemotherapy (median PFS 9.4 months with bevacizumab and chemotherapy versus 8.0 months with placebo plus chemotherapy).

Response assessment was based on radiological reports and/or clinical reports. Response was defined as evidence of tumor regression, stable disease as no change in tumor size, mixed response as regression in some tumors but progression in others, and progressive disease as tumor growth. All patients included in the study were newly diagnosed with stage IV CRC and had received no treatment for stage IV CRC. OS was calculated from diagnosis of metastatic disease until the date of death or censored at the last follow up date. Table 1 outlines the characteristics of patients included in the 2D-DIGE study.

**Table 1:** **Clinical features of patients in the 2D-DIGE discovery experiment**

Clinical features	Responders (n = 11)	Non responders (n = 12)
**Age** (range, years)	61 (47-74)	58 (29-71)
**Gender** (male/female)	7/4	4/8
**Site**		
Ascending colon	1 (9.%)	2 (16.7%)
Descending colon	3 (27.3%)	1 (8.3%)
Tranverse colon	0	1 (8.3%)
Sigmoid colon	5 (45.5%)	4 (33.3%)
Rectum	2 (18.2%)	4 (33.3%)
**Stage of CRC at diagnosis**		
Stage I	0	0
Stage II	0	0
Stage III	1 (9.1%)	0
Stage IV	10 (90.9%)	12 (100%)
**Differentiation**		
Well	1 (9.1%)	1 (8.3%)
Moderately	8 (72.7%)	5 (41.7%)
Poorly	1 (9.1%)	2 (16.7%)
Unknown	1 (9.1%)	4 (33.3%)
**Previous chemotherapy in Neoadjuvant/Adjuvant setting**		
Yes	0	0
No	11 (100%)	12 (100%)
**Chemotherapy for mCRC**		
FOLFOX/FLOX	4 (36.4%)	6 (50%)
FOLFIRI	2 (18.2%)	0
Xelox	4 (36.4%)	6 (50%)
5FU/Xeloda	1 (9%)	0
**Maintenance bevacizumab**		
Yes	4 (36.4%)	4 (33.3%)
No	7 (63.6%)	8 (66.7%)
**PFS**, median (range, days)	345 (301-720)	208 (93-260)
**Duration of bevacizumab treatment**, median, days, range	363 (138-880)	207 (83-460)

### Immunodepletion and sample preparation

Immunodepletion using the Multiple Affinity Removal System (MARS-14) was carried out as per manufacturer’s instructions (Agilent Technologies, Wilmington, DE, USA, 5188-6560). Serum (7 *μ*L) from each patient was diluted to 200 *μ*L with Buffer A (Agilent Technologies, Wilmington,DE, 5185-5987) and filtered through a 0.22 *μm* spin filter (Agilent, 5185-5990) for 1 min at 15 000 *g* to remove particulate matter. The diluted sample was placed into a MARS-14 spin cartridge. The spin cartridge was placed into a 1.5 mL collection tube, centrifuged for 1 min at 100 *g,* and the cartridge was let to sit for 5 min at room temperature. A further 400 *μ*L of buffer A was added to the cartridge and centrifuged for 2.5 min at 100 *g*. The spin cartridge was placed into a new collection tube, a further 400 *μ*L of buffer A was added, and then centrifuged for a further 2.5 min at 100 *g*. These two flow though fractions were combined. The flow though fraction comprised serum depleted of the 14 most highly abundant proteins. The spin cartridge was removed and 2.5 mL buffer B (Agilent, 5185-5988) was syringed through it in order to elute bound proteins. A further 5 mL of buffer A was syringed through the spin cartridge in order to re-equilibrate the cartridge. This process was repeated multiple times per sample in order to obtain adequate protein quantity for subsequent 2D-DIGE analysis.

Flow through fractions from individual patients samples were combined, placed into a spin concentrator with 5 KDa MWCO (Agilent, 5185-5991) and centrifuged at 3000 *g* at 10°C for 20 min. The retained fraction from the samples underwent precipitation using 4× volume of ice-cold acetone (Sigma-Aldrich, St Louis, Missouri, USA, 34850). The solution was incubated overnight at -20°C and then centrifuged at 15 000 *g* for 15 min at 4°C. Supernatants were discarded and protein pellets were resuspended in DIGE-specific lysis buffer (9.5 M urea, 2% CHAPS, 20 mM Tris, pH 8.5). To improve spot resolution from interfering salts, an Ettan 2-D Clean-Up Kit (GE Healthcare, Waukesha, WI, USA, 80-6484-51) was used. Pellets were resuspended in DIGE-specific lysis buffer. pH of samples were checked and optimised to a pH of 8.5. Protein concentration of the samples was determined with the Bradford assay as per the manufacturer’s instructions (Sigma-Aldrich).

#### Protein labelling

CyDyes were resuspended in anhydrous *N*, *N*-Dimethylformamide (DMF), 99.8% (Sigma-Aldrich, 227056) to give a stock solution of 1 mM and diluted prior to use with DMF to make a working solution of 400 pmol/μl. Individual depleted serum (50 *μ*g) samples were labelled with 400 pmol Cy3 (GE Healthcare, 25-8008-61). 50 *μ*g of each sample was pooled to make an internal standard and labelled with 400 pmol Cy5 (GE Healthcare, 25-8008-62). Labelling reactions were conducted on ice in the dark for 30 min and quenched by the addition of 1 *μ*L of 10 mM lysine (Sigma-Aldrich, L5626) for 10 minutes in the dark on ice. Following this, an equal volume of 2× dilution buffer (9.5 M urea, 2% CHAPS, 2% DTT, 1.6% Pharmalyte pH 3-10) was added to each sample. Individual labelled samples and the internal standard were then pooled and the total volume of the sample was made up to 450 *μ*L with rehydration buffer (8 M urea, 0.5% CHAPS, 0.2% DTT, 0.2% Pharmalyte pH 3-10).

#### Isoelectric focusing and SDS-PAGE

Each mixed sample underwent passive in-gel rehydration on Immobiline DryStrips pH 4-7, 24 cm (GE Healthcare, 17-6002-46) overnight in the dark. The strips were then focused using an Ettan IPGphor II (GE Healthcare) for 75,000 VHrs at 3,500 V with a holding step of 100 V. Following isoelectric focusing, each strip was equilibrated in a reducing buffer (6 M Urea, 50 mM Tris-HCl pH 8.8, 30% (v/v) glycerol, 2% (w/v) SDS, 1% (w/v) DTT) for 15 min followed by equilibration with an alkylating buffer (6 M Urea, 50 mM Tris-HCl, pH 8.8, 30% (v/v) glycerol, 2% (w/v) SDS, 4.8% (w/v) iodacetamide (IAA) for 15 min. The strips were placed on top of 12% SDS-PAGE gels and sealed with an agarose sealing solution (25 mM Tris, 192 mM glycine, 0.1% SDS, 0.5% (w/v) agarose, 0.02% Bromophenol blue). Protein separation in the second dimension was carried out at 1 W/gel in a PROTEAN Plus Dodeca Cell tank (Bio-Rad) at 15°C overnight in the dark in running buffer (25 mM Tris, 192 mM glycine, 0.1% SDS).

#### Image analysis

Gels were scanned upon completion of 2D electrophoresis with a Typhoon 9410 Variable Mode Imager (GE Healthcare). Photomultiplier for all images were kept within a range of 60,000 to 80,000 in order to decrease variation across gels. Final images were scanned at 100 *μm* pixel size and were cropped and exported into Progenesis Samespots v3.3 (Nonlinear Dynamics, UK). The accuracy of automated spot detection was confirmed by assessing the accuracy of the match vectors. Corrections to vector matching was performed by manual resetting using landmark points. Normalization and background subtraction was performed by the progenesis software. Statistically significant spots (ANOVA, p < 0.05, fold change ≥1.2) were identified, these parameters were similar to that used in other studies [13].

### Protein identification

Preparatory gels with approximately one milligram of pooled protein from depleted serum samples were run using the same 2DE conditions. Gels were fixed with 50% methanol and 10% acetic acid and then stained with PlusOne silver stain kit (GE Healthcare, 17-1150-01). Spots of interest were excised from the preparatory gels, destained, reduced, alkylated and digested with trypsin. The peptides were extracted three times with 50% ACN, 0.1% Trifluoroacetic acid (TFA) and resuspended in 0.1% TFA. The extracts were pooled and analysed using a LTQ-orbitrap XL mass spectrometer (Thermo Fisher Scientific, Rockford, IL, USA) connected to an Dionex Ultimate 3000 (RSLCnano) chromatography system (Dionex UK). Each sample was loaded onto Biobasic Picotip Emitter (120 mm length, 75 μm ID) packed with Reprocil Pur C18 (1.9 μm) reverse phase media column and separated by an increasing acetonitrile gradient using a 30 min reverse phase gradient at a flow rate of 300 nL/min. The mass spectrometer was operated in positive ion mode with a capillary temperature of 200°C, capillary Voltage 46 V, tube lens voltage 140 V and a potential of 1900 V applied to the frit. All data were acquired with the mass spectrometer operating in automatic data dependent switching mode. A high resolution MS scan (300-2000 Dalton) was performed using the Orbitrap to select the 7 most intense ions before MS/MS analysis using the ion trap.

#### Database search and protein identification

TurboSEQUEST (Bioworks Browser version 3.3.1 SP1; Thermo Finnigan, UK) was used to search the reviewed human subset of the Uniprot database, taxonomy (9606) for peptides cleaved with trypsin. Each peptide used for protein identification met specific SEQUEST parameters, i.e. a cross-correlation values of ≥1.9, ≥2.5, ≥3.2 and ≥3.2 for single-, double-, triple- and quadruple-charged peptides, respectively, and a peptide probability of <0.001 and 50% ion coverage. The observed spot migrations were compared to theoretical MW and pI values from the ExPASy Proteomics Server (Swiss Institute of Bioinformatics, Geneva).

### Gene Ontology and pathway analysis

Proteins that were identified as being differentially expressed were compared to annotated proteins by functional grouping based on gene ontology (GO) annotations using AMIGO [14] (v1.8) bioinformatics resource. Data were also analyzed through the use of Ingenuity Pathway Analysis (IPA) v9.0 (Ingenuity® Systems, http://www.ingenuity.com). A dataset containing Uniprot IDs and corresponding fold changes were uploaded into the application. Each identifier was mapped to its corresponding object in the Ingenuity® Knowledge Base application (application build-124019, content version-11631407). Only IPA networks with a score of 4 or greater, equivalent to a significance value of *p* < 0.0001, as used in other studies [15], were reported. These molecules, called Network Eligible molecules, were overlaid onto a global molecular network developed from information contained in the Ingenuity Knowledge Base. Networks of Network Eligible Molecules were then algorithmically generated based on their connectivity.

### Immunohistochemistry/ Elisa

Serum from 68 patients diagnosed with mCRC was collected prior to commencing chemotherapy and bevacizumab. Patient characteristics are described in Table 2. All patients included in the study were diagnosed with stage IV colorectal cancer at study entry. Tissue microarrays (TMAs) were constructed from 95 patients who had CRC surgery and prior to receiving chemotherapy and bevacizumab (Table 2).

**Table 2:** **Clinical features of patients in the validation experiments**

Clinical features	IHC patients (n = 95)	ELISA patients (n = 68)
**Age** (range, years)	67 (26-81)	61 (29-83)
**Gender** (male/female)	51/44	43/25
**Site**		
Ascending colon	32 (33.7%)	13 (19.1%)
Descending colon	1 (1%)	4 (5.9%)
Tranverse colon	0 (0%)	2 (2.9%)
Sigmoid colon	31 (32.6%)	25 (36.8%)
Rectum	31 (32.6%)	24 (35.3%)
**Stage of CRC at diagnosis**		
Stage I	1 (1%)	3 (4.4%)
Stage II	14 (14.7%)	8 (11.8%)
Stage III	34 (35.8%)	14 (20.6%)
Stage IV	46 (48.4%)	43 (63.2%)
**Differentiation**		
Well	5 (5.3%)	3 (4.4%)
Moderately	67 (70.5%)	39 (57.4%)
Poorly	19 (20%)	15 (22%)
Unknown	4 (4.2%)	11 (16.2%)
**Previous chemotherapy in Neoadjuvant/Adjuvant setting**		
Yes	33 (34.7%)	20 (29.4%)
No	62 (65.3%)	48 (70.6%)
**Chemotherapy for mCRC**		
FOLFOX/FLOX	35 (36.8%)	26 (38.2%)
FOLFIRI	15 (15.8%)	11 (16.2%)
Xelox	20 (21.1%)	22 (32.4%)
5FU/Xeloda	25 (26.3%)	9 (13.2%)
**Microsatellite instability**	7 (8%)	2 (3%)
Yes	64 (67%)	50 (73%)
No	24 (25%)	16 (24%)
Unknown		
**Maintenance bevacizumab**		
Yes	38 (40%)	25 (36.8%)
No	57 (60%)	43 (63.2%)
**PFS**, median (range, days)	340 (34-1655)	338 (43-1819)
**OS**, median (range, days)	784 (78-2110)	653 (98-1819)
**Duration of bevacizumab**, median, days (range)	242 (12-1169)	238 (12-1245)

### Immunohistochemistry

Four cores from two tumor blocks per patient were used for TMA analysis. 4 μm formalin fixed paraffin embedded (FFPE) sections were baked for 30 min at 90°C, deparaffinized in five changes of xylene, deionized water and then through graded alcohol concentrations. The deparaffinated sections were subjected to antigen retrieval in 6 M citrate buffer by microwaving. Incubation was performed overnight at 4°C with primary mouse monoclonal anti-apolipoprotein E (APOE), anti-angiotensinogen (AGT) and anti-vitamin D binding protein (DBP) (apolipoprotein E, Abcam 1907, 1:50 dilution; angiotensinogen, Abcam 86477, dilution 1:100; Vitamin D binding protein, Abcam 23485, dilution 1 μg/mL; Abcam, Cambridge, UK).

Following primary antibody incubation, endogenous peroxidase activity was blocked using 0.3% H2O2. Slides were incubated for 30 minutes with horseradish peroxidase–conjugated secondary antibody (Dako). Color was developed in diaminobenzidine solution (1:50; Dako) and counterstained with hematoxylin. Slides were mounted in pertex media. Tissue microarrays were scored for APOE, AGT, and DBP. The epithelium and stroma were scored as a percentage of the total cells in a blinded fashion according to the following system: 0%, 10%, 25%, 50%, 75%, 90% and 100% (Additional file 1: Figure S1). Scoring was performed by two investigators. If there was greater than 10% inter-observer variance, those cases were re reviewed and a consensus reached.

### ELISA

Enzyme linked immunosorbent assay (ELISA) was performed for APOE and AGT on serum from the original cohort, in addition to an independent group of patients (n = 68) with mCRC who had received bevacizumab treatment. ELISAs were performed in accordance with the manufacturer’s recommendations and included: Human apolipoprotein E (Mabtech, Sweden, 3712-1H-6) and Human Total Angiotensinogen Assay Kit (Immuno-Biological Laboratories, Japan, 27412).

### Statistics

PFS and OS were estimated by the Kaplan–Meier method for the patients included in the TMA and ELISA analysis. Statistically significant prognostic factors identified in univariate analyses were selected to enter multivariable analyses using a Cox proportional hazards model. A backwards elimination technique was used to select the final model, with a *p*-value less than 0.05 as the selection criteria. Hazard ratios (HRs) for TMA protein expression changes were calculated based on a ten percent change in protein expression. Statistical analyses were performed using SAS 9.2 (SAS Institute, Cary, NC).

Following multivariate analysis, proteins were divided into three subsets using the tertile points. These three subsets were classified as "Low", "Medium", and "High". For each subset, a product-limit survival estimate was obtained using the Kaplan-Meier method. Kaplan meier curves were constructed for illustrative purposes only.

## Results

### Biomarker discovery phase- 2D-DIGE analysis and LC-MS/MS protein identification

**Figure 2:**
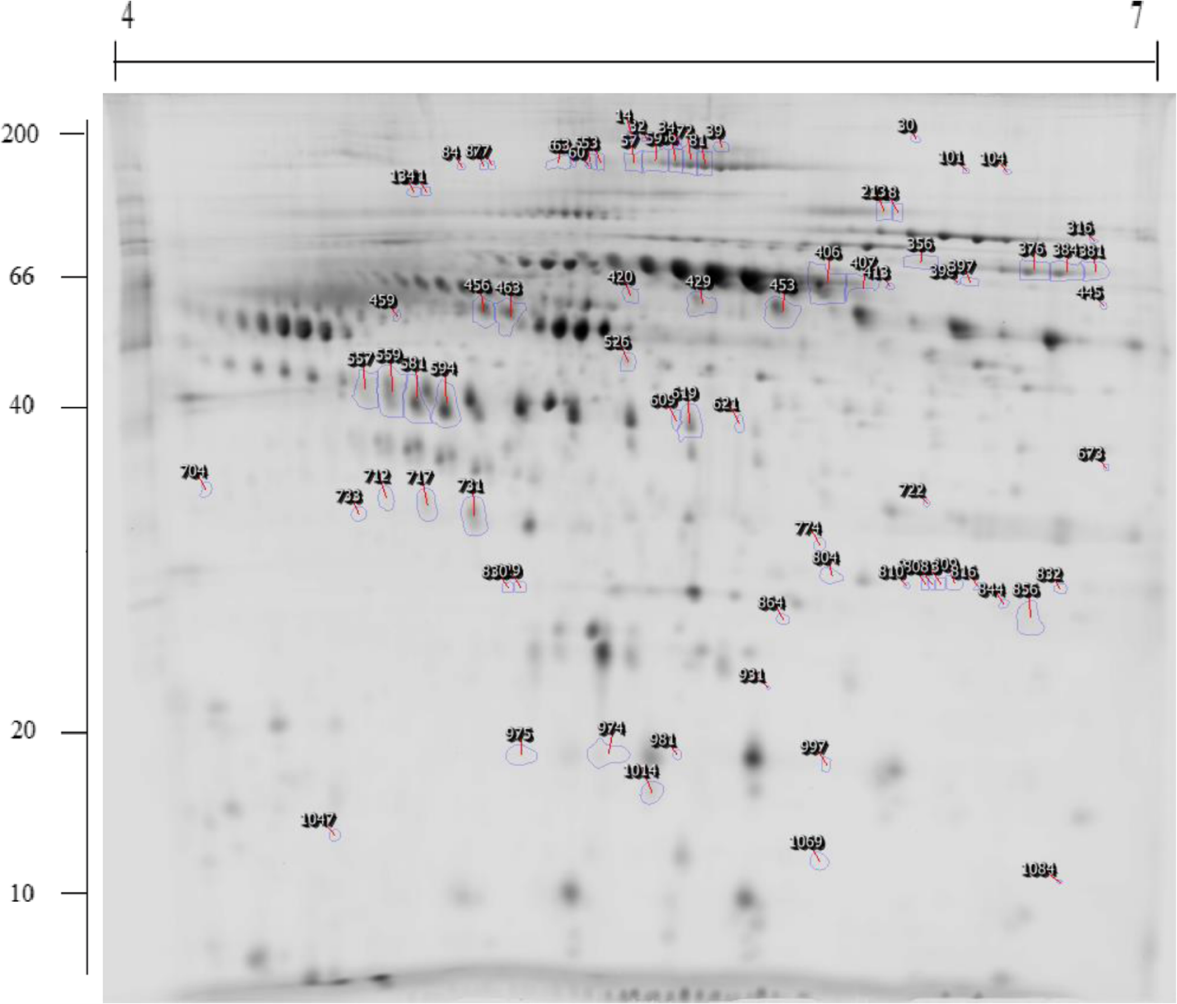
**Representative 2D-DIGE proteome map of serum from responders and non-responders to bevacizumab treatment.**

Approximately 1200 spots were detected on the 2D-DIGE gels. 80 spots displayed statistical significance (ANOVA, *p* < 0.05, fold change ≥1.2) between responders and non-responders (Figure 2). 51 statistically significant spots visible in the silver stained preparatory gels were excised, in-gel digested, analysed and identified using liquid chromatography-tandem mass spectrometry (LC-MS/MS) (Additional file 2: Table S1).

### Pathway analysis and gene ontologies

Following MS analysis, all successful protein identifications underwent functional classification by gene ontology using AMIGO. Overrepresented categories identified between the responding and non-responding patients included stress response, transport, signal transduction, immune system processes, structural development, cell death and catabolic processes, and cell differentiation (Additional file 3: Figure S2). This provided an indication of the functional relevance of the proteins identified following LC-MS/MS.

Literature searches also revealed that a number of the proteins isolated were known to influence the microenvironment of tumors. On the basis of these findings, three proteins were selected to go forward for validation APOE, AGT, and DBP.

In addition, we investigated network classifications, using IPA, to assess for interactions related to differentially expressed proteins in responders and non-responders (Additional file 4: Figure S3A, B, C). Proteins involved in cancer, gastrointestinal disease, and hepatic system disease, drug metabolism, molecular transport and lipid metabolism were the most significant networks observed.

### Protein validation

Protein expression data from 2D-DIGE demonstrated differential protein expression fold changes between responders and non-responders as follows: APOE- 1.65 fold, (*p* = 0.03); AGT- 3.45 fold, (*p* = 0.03); DBP-2.4 fold (*p* = 0.02).

#### ELISA

Serum concentrations of APOE and AGT were assessed by ELISA (Table 3). Increasing APOE serum levels showed a trend for shorter PFS (HR 1.17, 95% CI 0.99-1.37, *p* = 0.065) and OS (HR 1.17, 95% CI 0.99-1.39, *p* = 0.060). Increasing AGT concentration was associated with a significantly shorter OS (HR 1.12, 95% CI 1.03-1.21, *p* = 0.009).

**Table 3:** **Survival analysis and ELISA Analysis of serum proteins**

	Progression free survival					Overall survival			
Protein ^1^	p-value	Hazard ratio	95% Confidence interval		Protein ^1^	p-value	Hazard ratio	95% Confidence interval	
**APOE**	0.065	1.17	0.99	1.37	**APOE**	0.060	1.17	0.99	1.39
**AGT**	0.108	1.07	0.99	1.15	**AGT**	0.009	1.12	1.03	1.21
**Protein** ^**2**^	**p-value**	**Hazard ratio**	**95% Confidence interval**		**Protein** ^**2**^	**p-value**	**Hazard ratio**	**95% Confidence interval**	
**APOE**	0.065	1.17	0.99	1.37	**AGT**	0.009	1.12	1.03	1.21

^*1*^ *Univariate effects.*

^*2*^ *Model selected using Backwards Elimination procedure.*

#### Immunohistochemistry

All variables were assessed in a univariate analysis, by backwards elimination procedure, and in a multiple cox PH model for their association with PFS and OS. Increasing APOE stromal demonstrated a significantly shorter PFS and OS [(HR 1.34, 95% CI 1.10-1.63, p = 0.002), (HR 1.22, 95% CI 1.0-1.48, p = 0.036)] (Table 4), respectively. This remained significant following a backwards elimination procedure. However increasing APOE epithelial expression demonstrated a longer PFS (HR 0.90, 95% CI 0.82-1.0, p = 0.011) and OS. This remained significant following a backwards elimination procedure. Increasing DBP stromal expression demonstrated a significantly shorter OS (HR 1.22 95% CI 1.0-1.34, p = 0.037) in univariate analysis and following a backwards elimination procedure.

**Table 4:** **Univariate, backwards elimination and multiple cox PH model of proteins assessed by IHC**

PFS									
Variable	Univariable			Backwards elimination			Multiple Cox PH model		
	HR ^a^	95% CI	p-value	HR ^a^	95% CI	p-value	HR ^a^	95% CI	p-value
**APOE epithelium**	0.90	0.82-1.0	0.011	0.82	0.74-0.90	0.0007	0.82	0.74-0.90	0.0007
**APOE stroma**	1.34	1.10-1.63	0.002	1.48	1.22-1.79	0.0001	1.48	1.22-1.79	0.0001
**AGT epithelium**	0.90	0.82-1.0	0.006	0.90	0.82-1.0	0.006			
**AGT stroma**	0.90	0.82-1.10	0.579						
**DBP epithelium**	1.00	0.90-1.10	0.753						
**DBP stroma**	1.10	0.90-1.34	0.201						
**OS**									
**APOE epithelium**	0.90	0.82-1.0	0.179	0.90	0.82-1.0	0.043	0.90	0.82-1.0	0.04
**APOE stroma**	1.22	1.0-1.48	0.036	1.22	1.10-1.48	0.012	1.22	1.10-1.48	0.01
**AGT epithelium**	0.90	0.82-1.0	0.90						
**AGT stroma**	1	0.90-1.22	0.67						
**DBP epithelium**	1.0	0.90-1.10	0.58						
**DBP stroma**	1.22	1.0-1.34	0.037	1.22	1.0-1.34	0.037			

^a^Hazard ratio were calculated based on a ten percent change in protein expression.

*Abbreviations*: *AGT* angiotensinogen, *APOE* apolipoprotein E, *DBP* vitamin D binding protein.

Increasing expression of epithelial AGT demonstrated a significant improvement in PFS (HR 0.90, 95% 0.82-1.00, p = 0.006) in the univariate analysis, and this remained significant following a backwards elimination procedure. However, there was no significance demonstrated between epithelial AGT and OS. When proteins were combined in a multiple cox PH model, increasing APOE stromal expression remained significant for shorter PFS (p = 0.001) and OS (p = 0.01). Furthermore, increasing epithelial APOE expression remained significant for a longer PFS (p = 0.0007) and OS (p = 0.04) in a multiple PH model.

**Figure 3:**
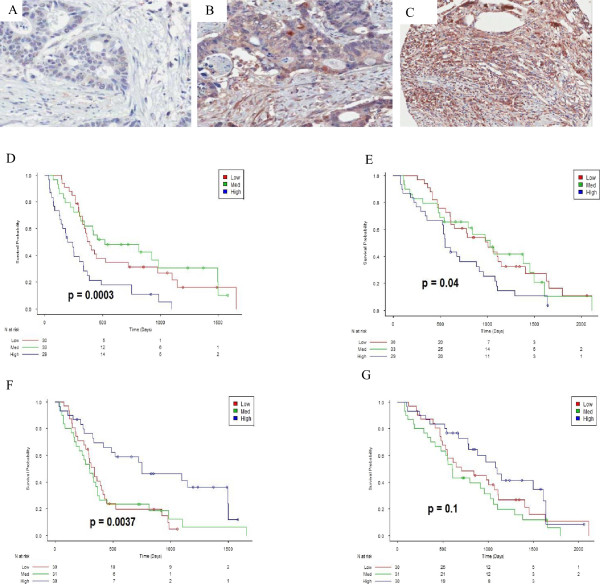
**Survival and APOE expression.** Representative images of APOE expression demonstrating **(A)** low, **(B)** medium and **(C)** high expression, **(D)** PFS Kaplan meier curve of APOE stromal expression of high, medium and low expression, demonstrating significantly shorter PFS in patients with high expression, **(E)** OS Kaplan meier curve of APOE stromal expression of high, medium and low expression, demonstrating significantly shorter OS in patients with high expression, **(F)** PFS Kaplan meier curve of APOE epithelial expression of high, medium and low expression, demonstrating significantly longer PFS in patients with high expression, **(G)** OS Kaplan meier curve of APOE epithelial expression of high, medium and low expression, demonstrating no significance between between the groups.

Proteins were divided into three subsets using the tertile points. These three subsets were classified as "Low", "Medium", and "High". For each subset, a product-limit survival estimate was obtained using the Kaplan-Meier method.‘High’ APOE stromal expression demonstrated a significantly shorter PFS and OS compared with medium and low expression. (Figure 3D,E). Conversely, ‘high’ APOE (epithelial) expression demonstrated a significantly longer PFS than medium and low expression (Figure 3F), however no significance was seen between the three groups for OS (Figure 3G).

**Figure 4:**
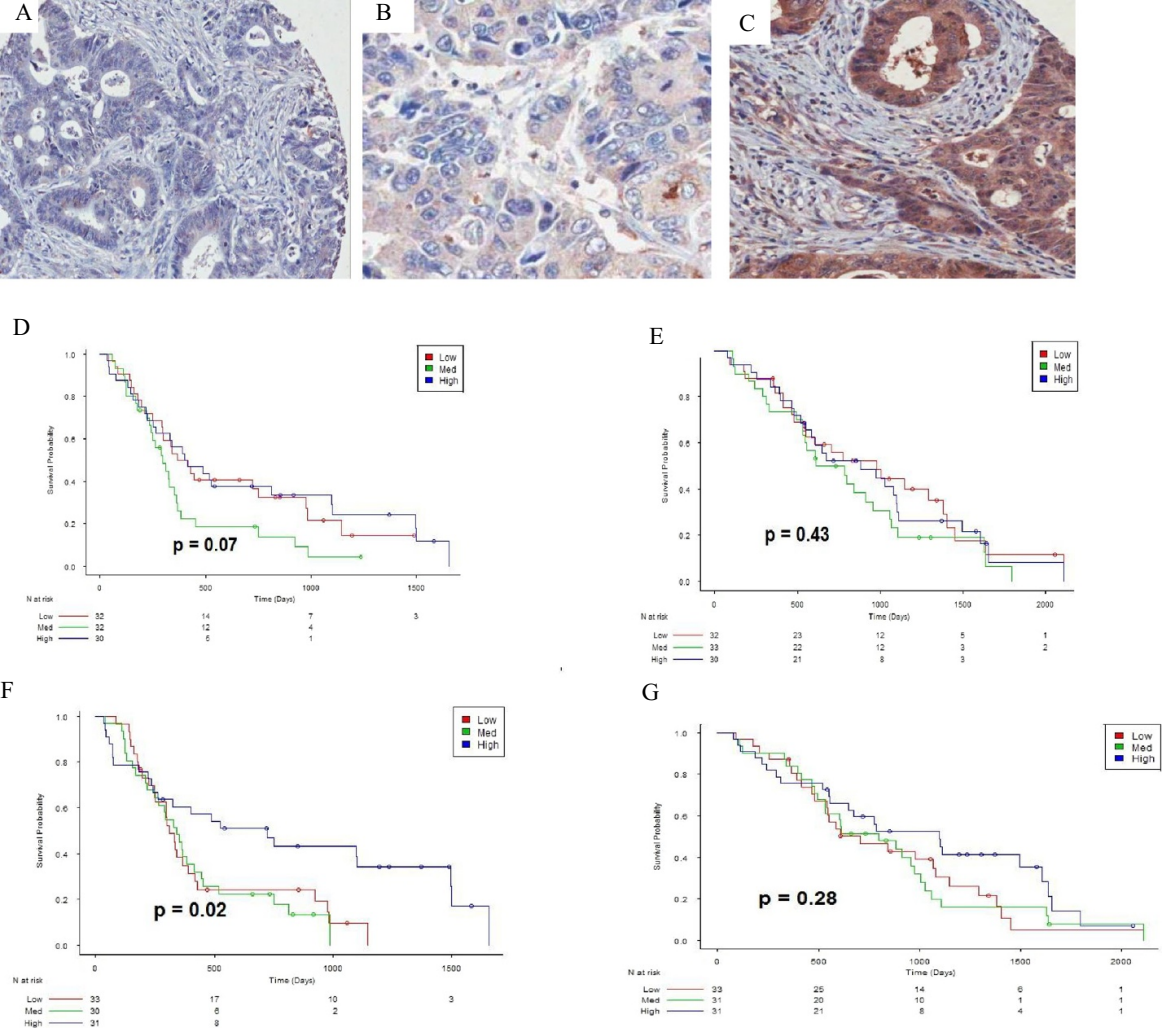
**Survival and AGT expression.** Representative images of AGT expression demonstrating **(A)** low, **(B)** medium and **(C)** high expression, **(D)** PFS Kaplan meier curve of AGT stromal expression of high, medium and low expression, demonstrating no effect of the three groups on PFS **(E)** OS Kaplan meier curve of AGT stromal expression of high, medium and low expression, demonstrating no effect of the three groups on OS **(F)** PFS Kaplan meier curve of AGT epithelial expression of high, medium and low expression, demonstrating significantly longer PFS in patients with high expression, **(G)** OS Kaplan meier curve of AGT epithelial expression of high, medium and low expression, demonstrating no effect of the three groups on OS.

There was no effect of the three groups on PFS or OS for stromal AGT expression (Figure 4D,E). High epithelial AGT expression demonstrated a significantly longer PFS than medium and low expression (Figure 4F), however no significant effect of the three groups on OS was seen (Figure 4G).

**Figure 5:**
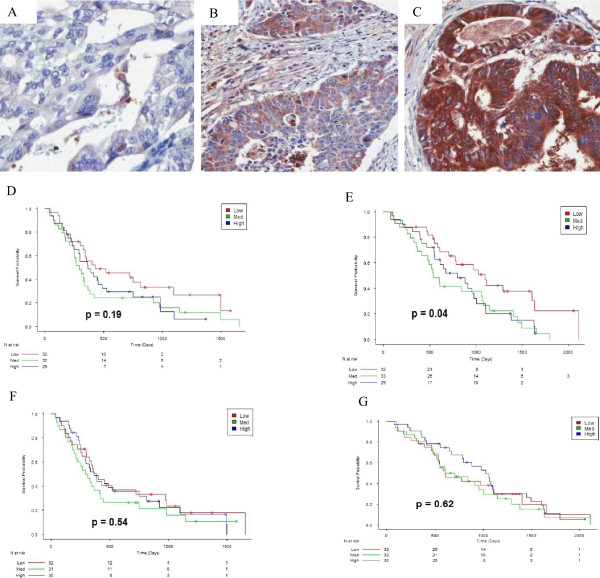
**Survival and DBP expression.** Representative images of DBP expression demonstrating **(A)** low, **(B)** medium and **(C)** high expression, **(D)** PFS Kaplan meier curve of DBP stromal expression of high, medium and low expression, demonstrating no effect of the three groups on PFS **(E)** OS Kaplan meier curve of DBP stromal expression of high, medium and low expression, demonstrating a significantly longer OS in patients with low expression, **(F)** PFS Kaplan meier curve of DBP epithelial expression of high, medium and low expression, demonstrating no effect of the three groups on PFS **(G)** OS Kaplan meier curve of DBP epithelial expression of high, medium and low expression, demonstrating no effect of the three groups on OS.

Low expression of stromal DBP demonstrated a significantly longer OS than medium and high expression (Figure 5E), however no differences were seen for PFS (Figure 5D).

There was no distinguishable difference between the high, medium and low groups for PFS and OS for epithelial DBP (Figure 5F,G).

## Discussion

Identifying patients who will respond to a given targeted therapy is a key factor in delivering personalised medicine. Biomarkers hold the potential to identify patients who may benefit from a treatment, detect cancer at an early stage and avoid unnecessary toxicity for patients who are unlikely to respond. No biomarkers are currently known that can identify patients who will respond to bevacizumab.

In our initial 2D-DIGE discovery study on depleted serum we identified differential protein expression between the two groups of patients. Candidate biomarkers were selected for validation for their potential functional relevance and literature searches which demonstrated the proteins to have an association with a range of malignancies. There were limitations in this study which included no group of patients that did not receive bevacizumab, and therefore identified markers may be predictive of response to chemotherapy or bevacizumab.

APOE is a 299 amino acid glycoprotein with a molecular mass of approximately 34,000 KDa [16]. Its role in regulating lipid metabolism is well known, however it is increasingly being recognised to have other functions including antioxidant effects, immune activity, cell signalling, inhibitor of proliferation of several cell types, modulation of angiogenesis and tumor growth [17, 18]. In addition, APOE has been shown to play a role in many cancer types [19–22].

In our study, increasing APOE serum levels demonstrated a trend for shorter PFS and OS in univariate analysis. Following backwards elimination, this trend remained for PFS. Increasing APOE stromal expression was associated with shorter PFS and OS, whereas increasing APOE epithelial expression was associated with a longer PFS and OS. This discrepancy between epithelium and stromal subcomponents may reflect that patterns of expression often differ between epithelium and stromal cells and have differential response to signals that modulate proliferation and/or apoptosis [23]. It has been recognised that disruption of the homeostatic interactions between epithelium and stroma could initiate and promote carcinogenesis.

One study evaluating the significance of APOE expression in gastric cancer, found that APOE mRNA was more highly expressed in gastric cancer tissue than corresponding normal mucosa [21]. Immunohistochemistry showed that APOE was predominantly expressed in gastric cancer. Furthermore, patients with high APOE tumor expression had a shorter survival than those with low APOE expression. APOE has also been studied in prostate cancer and expression varies with the Gleason score, suggesting that APOE expression may represent a marker of more aggressive tumors [19].

APOE has been investigated in CRC and it has been proposed that it may play a role in the development of CRC by three mechanisms- cholesterol and bile metabolism, triglyceride and insulin regulation, and inflammation [22]. In addition, APOE has been shown to be a potent inhibitor of the proliferation of several cell types and may be effective in modulating angiogenesis and tumor cell growth [24]. ApoEdp, a dimer peptide derived from the receptor binding region of APOE, has demonstrated significant inhibition of human breast xenografts which were implanted into nude mice compared with PBS [24]. ApoEdp also demonstrated anti-angiogenic effects by inhibiting VEGF-induced angiogenesis in a rabbit eye model [24]. ApoEdp selectively blocked VEGF-induced Flk-1 receptor activation and the downstream angiogenic signalling pathway of c-Src-Akt-eNOS, FAK, and Erk1/2 which promote tumor development. Although further investigation into the anti-angiogenic tumor properties of APOE is required in different cancer models, these results pose interesting theories regarding the pharmacological anti-angiogenic activity of APOE.

DBP is a plasma carrier protein of vitamin D compounds with a molecular weight of approximately 52-59 kDa [25]. In our study, increasing vitamin D stromal expression was associated with poorer OS in both univariate analysis and following a backwards elimination procedure.

DBP has been identified as a biomarker in several cancers including breast, oral, pancreas and lung cancer [26–29]. The significance of circulating DBP levels with regards to vitamin D’s biologic action was investigated in one study where it was found that measured levels of 25-hydroxyvitamin D (25(OH)D) and DBP levels were positively correlated leading to speculation that total circulating levels of 25(OH)D may be determined in part by DBP levels [30]. Therefore, the actions of DBP and vitamin D and its related compounds are interconnected. Epidemiological studies have supported a link between vitamin D and colorectal risk [31–34]. However, the role of DBP in colonic carcinogenesis is less well characterised. It has been hypothesised that DBP may play a role in malignancy due to its role as a precursor for the macrophage-activating factor, its function as an actin scavenger, and via its anti-angiogenic properties [29, 35, 36]. DBP is a precursor for the macrophage-activating factor (maf) and is converted to DBP-maf [35, 37]. DBP-maf has anti-angiogenic activity [37, 38], in addition to activating macrophages to aid in the eradication of cancer [35]. Further investigation in prostate cancer cell lines demonstrated that DBP-maf had strong inhibitory activity independent of macrophage activation [39].

The effects of 1α, 25-dihydroxyvitamin D_3_ [1,25(OH)_2_D_3_], the active form of the vitamin D, on angiogenesis and cancer have been investigated [40]. The administration of 1,25(OH)_2_D_3_ was shown to have anti-angiogenic properties in human cancer cell lines including prostate, breast and colon, with resulting inhibition of VEGF secretion under both normoxic and hypoxic conditions. Angiogenesis is stimulated in response to hypoxia and this is mediated by hypoxic-inducible factor (HIF)-1. The treatment of prostate and colon cell lines with 1,25(OH)_2_D_3_ under hypoxic conditions resulted in reduced levels of HIF-1α and HIF target genes.

Another study examin ed the effect of the administration of vitamin D3-related compounds 1α (OH)D3 and 1,25(OH)_2_D3 on the incidence of colon tumors in Wistar rats induced by azoxymethane, and this demonstrated inhibition of the development of colon tumors [41]. This may be related to the inhibition of angiogenesis as prolonged administration of 1α(OH)D3 and 1,25(OH)_2_D_3_ significantly decreased vessel count and decreased immunohistochemical staining of VEGF.

AGT, a member of the serpin family, was also validated in both serum and tissue in our study. Increasing serum levels were significantly associated with worse OS, and epithelial expression of AGT was significantly associated with improved PFS.

AGT, with an approximate molecular weight of 56,800, is synthesised mainly in the liver [42]. AGT has been shown to have anti-angiogenic properties [43, 44] and block the formation of capillary like structures *in vitro* (capillary-like tube formation on Matrigel) and *in vivo* (the chick chorioallantoic membrane assay) [43, 45]. The angiogenic and tumor growth effects of human AGT were further investigated *in vivo* in a transgenic mouse model. Transgenic mice expressing human AGT were crossed with a transgenic mouse model of hepatocellular carcinoma [42]. The bitransgenic mice overexpressing AGT had longer survival time, reduction of tumour growth and blood flow velocities in the liver compared with the hepatocellular model. The bitransgenic mice demonstrated reduced angiogenesis, impaired expression of endothelial arterial markers and decreased arterial vessel density, thereby providing evidence that AGT displays anti-angiogenic tumor properties. The effect that anti-angiogenic targeted therapy has on AGT remains unclear. In addition, the interaction between circulating levels of AGT and tissue expression has not been clearly defined.

## Conclusion

Identifying a sole biomarker which is able to identify patients who respond to a treatment may be difficult, and it may be that a panel of markers may provide a more reliable assessment of response.

We have confirmed the differential expression of APOE, AGT, and DBP in the original samples and within an independent series of serum and tissue samples in patients treated with bevacizumab and chemotherapy for mCRC. However characterisation of these proteins in a larger cohort of patients will be required before any firm conclusions regarding their application as potential markers can be made.

## Electronic supplementary material

Additional file 1: Figure S1: Representative image demonstrating AGT expression in stroma and epithelium. (PPT 352 KB)

Additional file 2: Table S1: LC-MS/MS data for differentially expressed spots between responders and non-responders to bevacizumab. (DOC 328 KB)

Additional file 3: Figure S2: Gene Ontology (GO) functional classification. Biological process of differentially expressed proteins identified between responders and nonresponders. Many serum proteins are multi-functional and therefore proteins may be found in more than one functional group. The numbers listed on the diagram represent the number of proteins in that functional group. (PPT 542 KB)

Additional file 4: Figure S3: **A**, **B**, **C**: Ingenuity pathway interaction network analysis of proteins differentially expressed between responder and non-responder groups. **(A)** Network 1, proteins involved in cancer, gastrointestinal Disease and Hepatic System Disease; **(B)**. Network 2, proteins involved in drug metabolism, molecular transport and lipid metabolism. **(C)** The network displays nodes (genes/gene products) and edges (the biological relationship between nodes). The color intensity of the nodes indicates the fold change (red: increase; green: decrease) associated with a particular protein in serum from the responder compared with the non-responder group. A solid line indicates a direct interaction between nodes (genes/gene products) and a dashed line indicates an indirect relationship between nodes. The shape of the node is indicative of its function. (PPT 795 KB)

## Abbreviations

mCRC: Metatstatic colorectal cancer
APOE: Apoliprotein E
AGT: Angiotensionogen
DBP: Vitamin D binding protein
VEGF-A: Vascular endothelial growth factor-A
PFS: Progression free survival
OS: Overall survival
mRNA: Messenger RNA.
