# Supplementary figures and images for: Predicting response to vascular endothelial growth factor inhibitor and chemotherapy in metastatic colorectal cancer
Source: BMC Cancer. 2014 Nov 27;14:887. doi: 10.1186/1471-2407-14-887 (PMC4289341; doi:10.1186/1471-2407-14-887)

## Slide 1
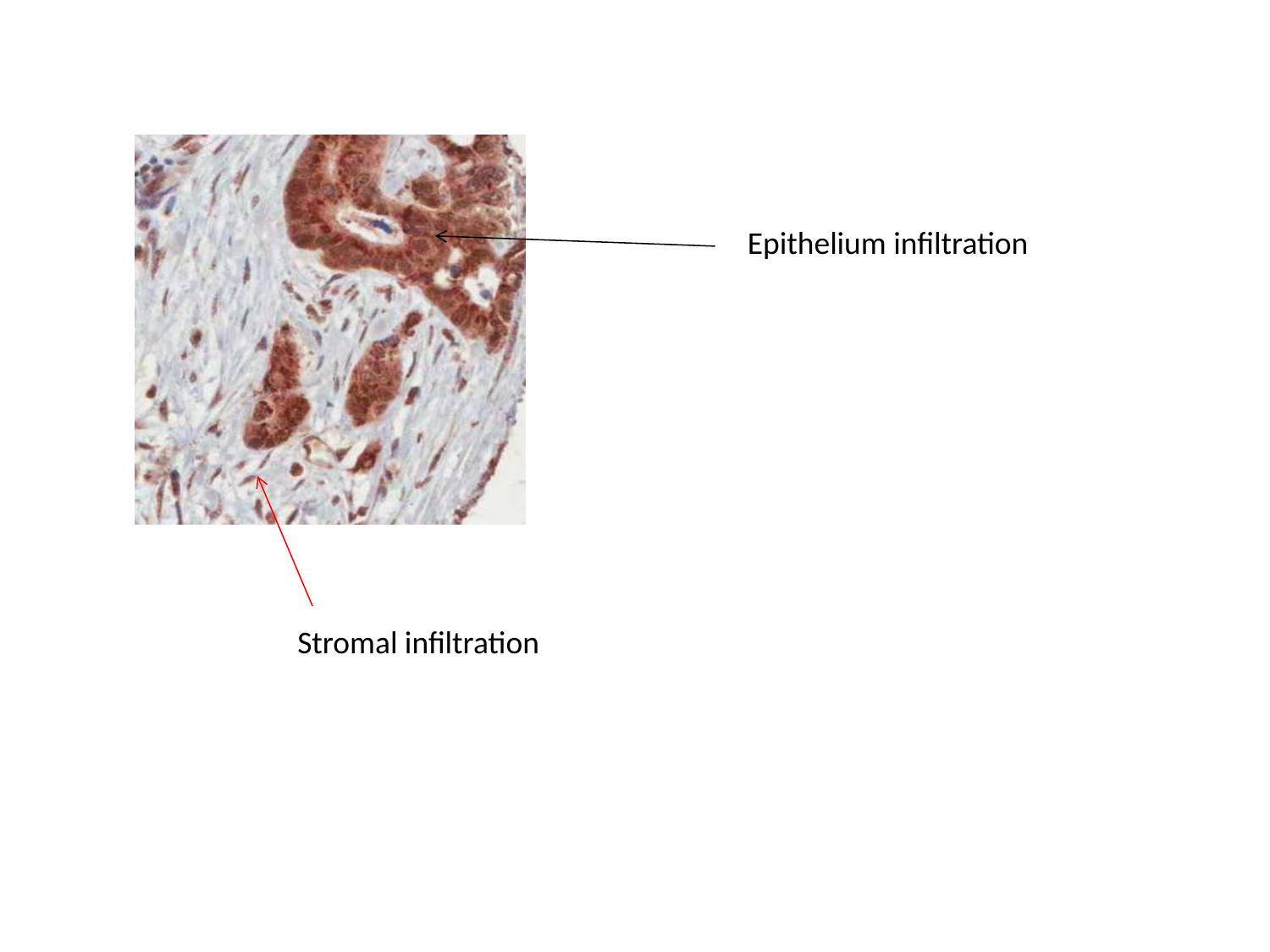

Epithelium infiltration
Stromal infiltration

Supplement: Supplementary file 1 — Additional file 1: Figure S1: Representative image demonstrating AGT expression in stroma and epithelium. (PPT 352 KB) [file 12885_2014_5131_MOESM1_ESM.ppt]

## Slide 1
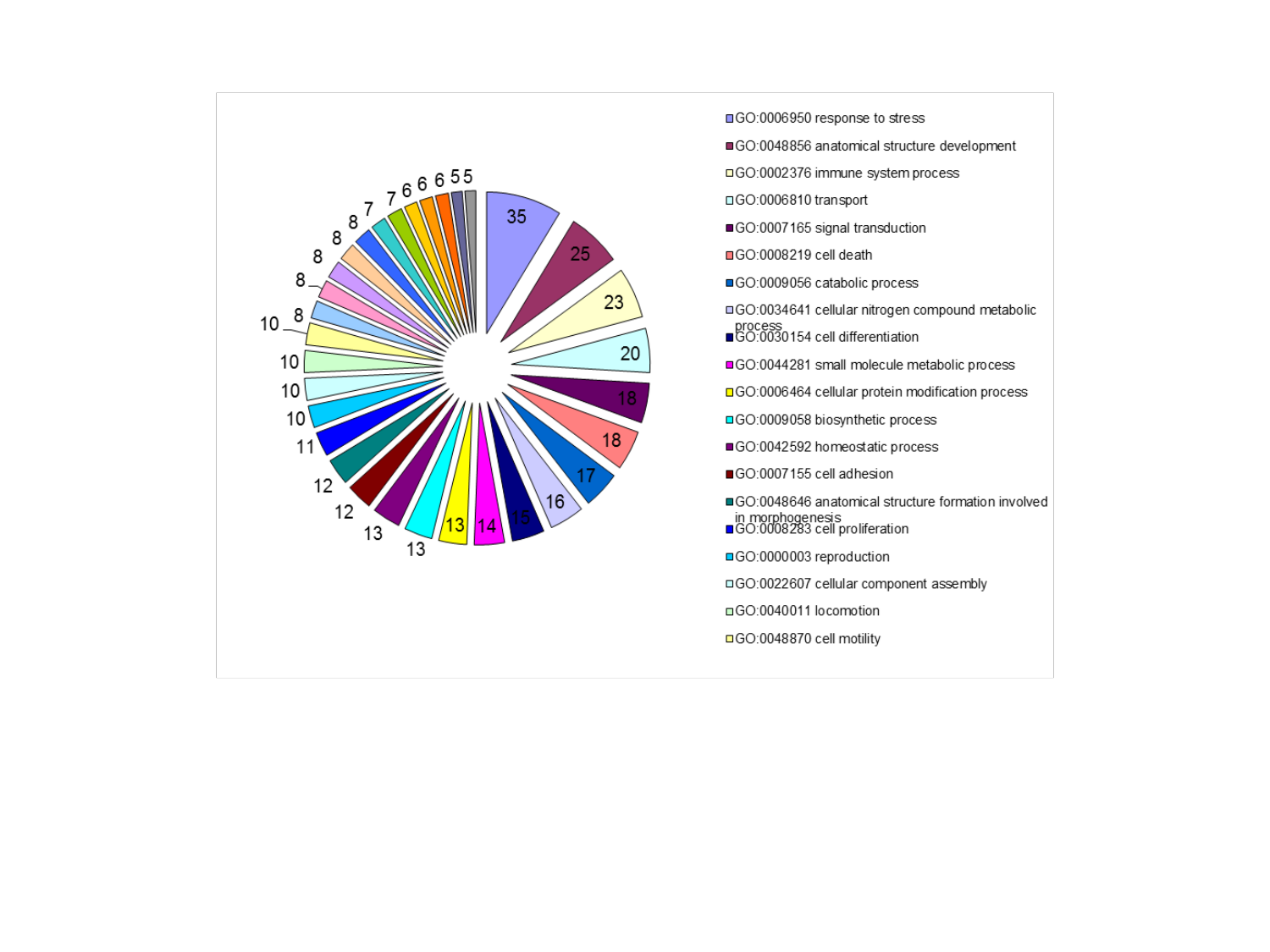

Supplement: Supplementary file 3 — Additional file 3: Figure S2: Gene Ontology (GO) functional classification. Biological process of differentially expressed proteins identified between responders and nonresponders. Many serum proteins are multi-functional and therefore proteins may be found in more than one functional group. The numbers listed on the diagram represent the number of proteins in that functional group. (PPT 542 KB) [file 12885_2014_5131_MOESM3_ESM.ppt]

## Slide 1
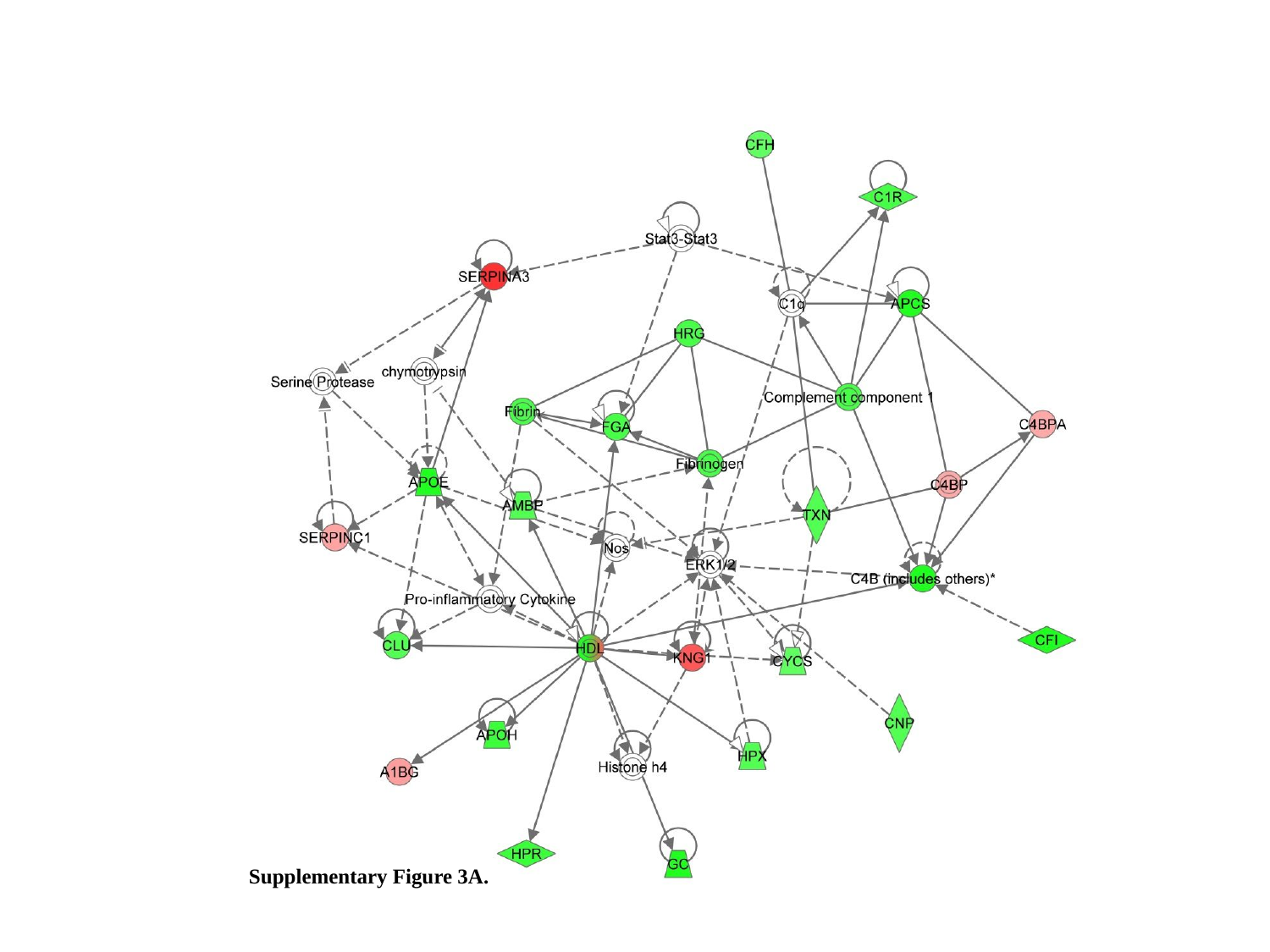

Supplementary Figure 3A.

## Slide 2
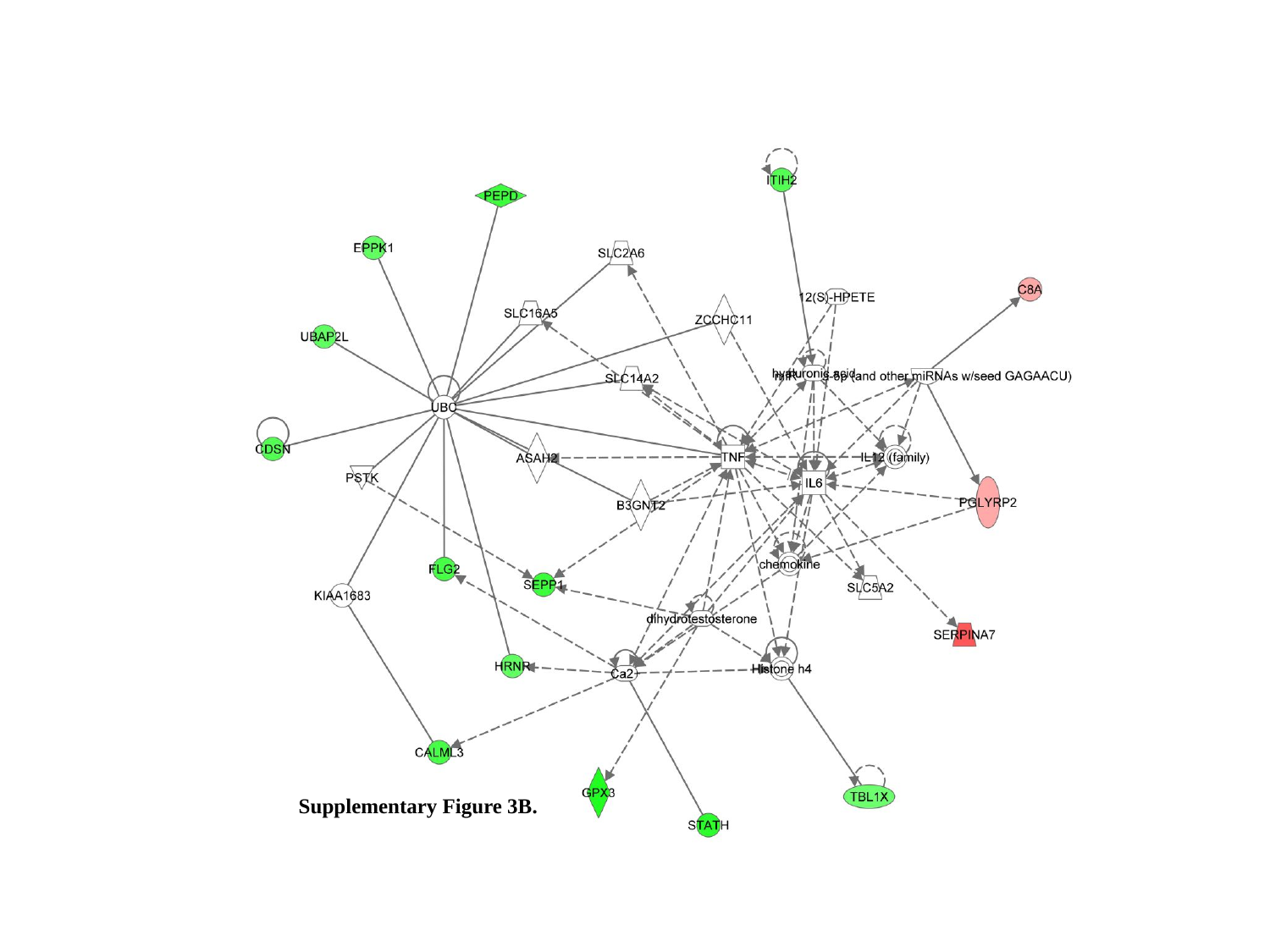

Supplementary Figure 3B.

## Slide 3
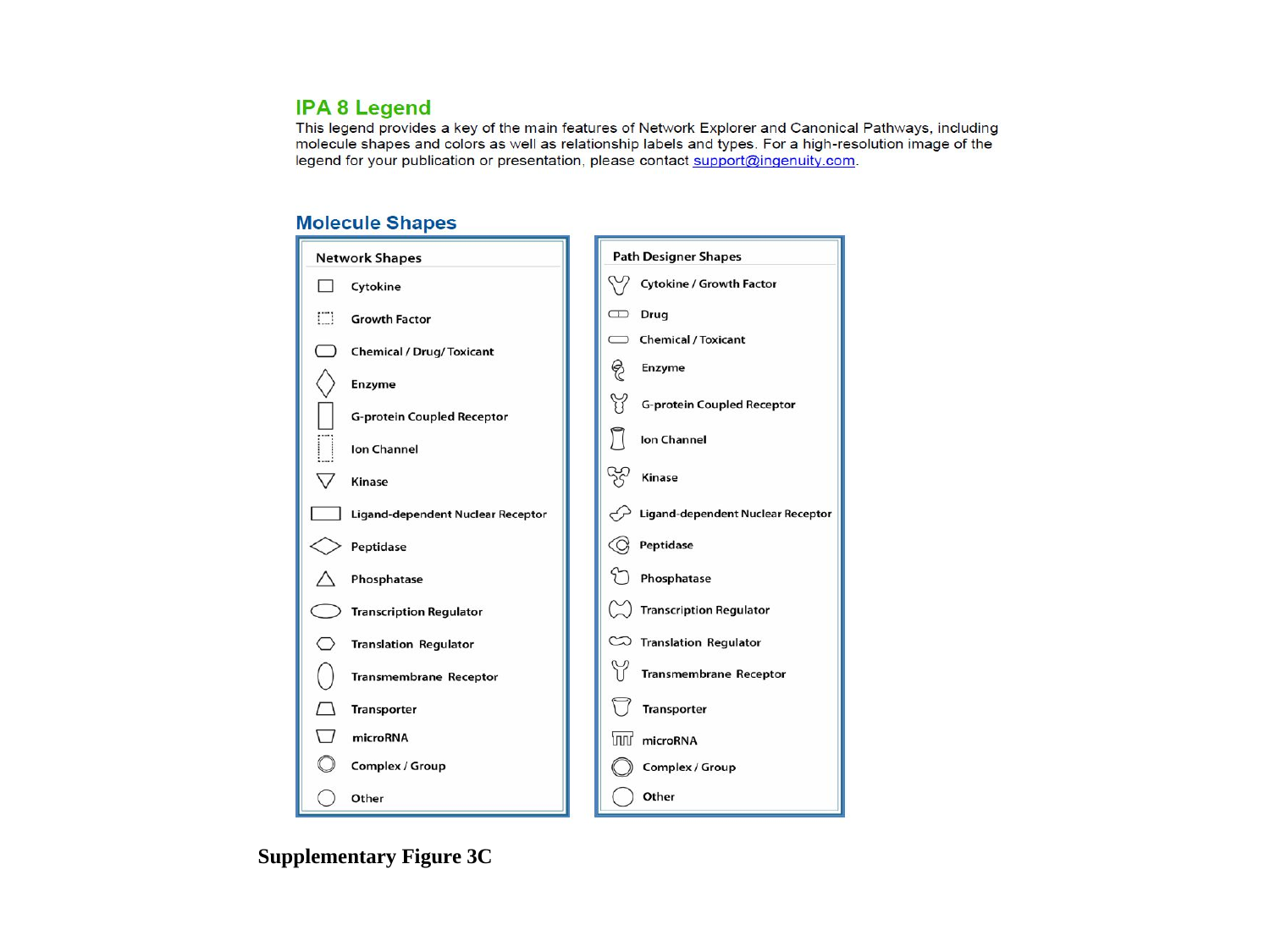

Supplementary Figure 3C

Supplement: Supplementary file 4 — Additional file 4: Figure S3: A, B, C: Ingenuity pathway interaction network analysis of proteins differentially expressed between responder and non-responder groups. (A) Network 1, proteins involved in cancer, gastrointestinal Disease and Hepatic System Disease; (B). Network 2, proteins involved in drug metabolism, molecular transport and lipid metabolism. (C) The network displays nodes (genes/gene products) and edges (the biological relationship between nodes). The color intensity of the nodes indicates the fold change (red: increase; green: decrease) associated with a particular protein in serum from the responder compared with the non-responder group. A solid line indicates a direct interaction between nodes (genes/gene products) and a dashed line indicates an indirect relationship between nodes. The shape of the node is indicative of its function. (PPT 795 KB) [file 12885_2014_5131_MOESM4_ESM.ppt]
