# Supplementary material for: Predicting response to vascular endothelial growth factor inhibitor and chemotherapy in metastatic colorectal cancer
Source: BMC Cancer. 2014 Nov 27;14:887. doi: 10.1186/1471-2407-14-887 (PMC4289341; doi:10.1186/1471-2407-14-887)
Supplement: Supplementary file 2 — Additional file 2: Table S1: LC-MS/MS data for differentially expressed spots between responders and non-responders to bevacizumab. (DOC 328 KB) [file 12885_2014_5131_MOESM2_ESM.doc]

| **Accession no.**  **Additional file 2: Table S1 LC-MS/MS data for differentially expressed spots between responders and non-responders to bevacizumab** | **Protein ID** | **Fold change** | **Direction of change, Non-responder: Responder** | **Spot Number^a^** | **No. Peptides** | **MW Observed** | **PI Observed** | **MW Theoretical^b^** | **PI Theoretical^b^** | **Score XC^c^** | **Seq. Coverage %** | **P-value** |
| --- | --- | --- | --- | --- | --- | --- | --- | --- | --- | --- | --- | --- |
| P62736 | Actin, aortic smooth muscle | 1.4 | **↓** | 717 | 1 | 34000 | 4.83 | 41747.76 | 5.24 | 10.14 | 4.24 | 0.012 |
| P01011 | Alpha-1-antichymotrypsin | 3.8 | **↑** | 456 | 1 | 58000 | 5.01 | 45265.82 | 5.32 | 10.14 | 4.26 | 0.03 |
| P04217 | Alpha-1B-glycoprotein | 1.6 | **↑** | 384 | 3 | 65000 | 6.75 | 51921.66 | 5.63 | 30.18 | 8.69 | 0.03 |
| P06733 | Alpha-enolase | 1.2 | **↓** | 72 | 1 | 135000 | 5.63 | 47037.77 | 6.99 | 10.15 | 2.76 | 0.007 |
|  |  | 1.2 |  | 57 | 1 | 140000 | 4.94 |  |  | 10.21 | 5.07 | 0.03 |
| P02743 | amyloid P-component | 1.7 | **↓** |  |  | 59000 | 5.65 | 23258.53 | 6.12 | 30.18 | 17.04 | 0.03 |
|  |  | 1.6 |  | 429 | 3 |  |  |  |  |  |  |  |
|  |  |  |  | 619 | 3 | 40000 | 5.63 |  |  | 30.17 | 15.7 | 0.04 |
| P01019 | Angiotensinogen | 3.8 | **↑** | 456 | 1 | 57500 | 5.05 | 49729.69 | 5.6 | 10.18 | 3.51 | 0.03 |
|  |  | 3.1 |  | 463 | 1 | 57000 | 5.08 |  |  | 10.14 | 2.68 | 0.018 |
| P01008 | Antithrombin-III | 3.8 | **↑** | 456 | 3 | 57500 | 5.04 | 49039.14 | 5.95 | 30.19 | 8.62 | 0.03 |
|  |  | 1.7 |  | 429 | 1 | 59000 | 5.65 |  |  | 10.13 | 2.37 | 0.03 |
|  |  | 3.1 |  | 463 | 1 | 57000 | 5.08 |  |  | 10.16 | 2.8 |  |
| P02649 | Apolipoprotein E | 1.7 | **↓** | 429 | 1 | 59000 | 5.65 | 36172.1 | 5.52 | 10.15 | 4.73 | 0.03 |
|  |  | 1.6 |  | 619 | 2 | 40000 | 5.63 |  |  | 20.19 | 9.46 | 0.04 |
| P05089 | Arginase-1 | 1.2 | **↓** | 72 | 1 | 135000 | 5.63 | 34734.94 | 6.72 | 10.16 | 6.21 | 0.007 |
| P02749 | Beta-2-glycoprotein | 1.5 | **↓** | 453 | 19 | 58000 | 5.89 | 36254.6 | 8.37 | 190.32 | 49.86 | 0.025 |
|  |  | 1.7 |  | 429 | 17 | 59000 | 5.65 |  |  | 170.29 | 42.32 | 0.03 |
|  |  | 1.3 |  | 731 | 1 | 34000 | 4.97 |  |  | 10.16 | 4.06 | 0.03 |
| P04003 | C4b-binding protein alpha chain |  | **↑** | 406 | 3 | 61000 | 6.02 | 61630.43 | 6.24 | 30.19 | 6.9 | 0.028 |
|  |  | 1.6 |  |  |  |  |  |  |  |  |  |  |
| P27482 | Calmodulin-like protein 3 | 1.4 | **↓** | 717 | 1 | 34000 | 4.83 | 16759.52 | 4.3 | 10.14 | 11.41 | 0.012 |
|  |  |  |  |  |  |  |  |  |  |  |  |  |
| Q9NZT1 | Calmodulin-like protein 5 | 1.4 | **↓** | 717 | 1 | 34000 | 4.83 | 15761.32 | 4.31 | 10.23 | 15.75 | 0.012 |
| P00450 | Ceruloplasmin | 1.2 | **↓** | 57 | 11 | 140000 | 5.47 | 120085.49 | 5.41 | 110.3 | 14.18 | 0.012 |
|  |  | 1.3 |  | 59 | 6 | 139000 | 5.55 |  | 5.55 | 60.3 | 9.67 | 0.004 |
|  |  | 1.2 | **↓** | 72 | 1 | 135000 | 5.63 |  | 5.63 | 10.17 | 1.97 | 0.007 |
| P10909 | Clusterin | 1.3 |  | 731 | 1 | 34000 | 4.97 | 50062.56 | 5.89 | 10.16 | 10.16 | 0.03 |
|  |  | 1.4 | **↓** | 717 | 1 | 34000 | 4.83 |  |  | 10.17 | 3.79 | 0.012 |
| P0C0L4 | Complement C4-A | 1.4 |  | 581 | 1 | 44000 | 4.8 | 84183.32 | 5.33 | 10.13 | 0.46 | 0.014 |
|  |  | 1.7 | **↓** | 429 | 6 | 59000 | 5.65 |  |  | 60.19 | 4.01 | 0.03 |
| P00736 | Complement C1r subcomponent |  | **↓** | 594 | 1 | 43000 | 4.98 | 78213.16 | 5.76 | 10.19 | 1.99 | 0.023 |
|  |  | 1.4 |  |  |  |  |  |  |  |  |  |  |
| P0C0L5 | Complement C4-B | 1.7 | **↓** | 429 | 1 | 59000 | 5.65 | 71678.89 | 8.69 | 10.29 | 1.55 | 0.03 |
| P07357 | Complement component C8 alpha chain | 1.6 | **↓** | 406 | 3 | 61000 | 6.02 | 61711.04 | 5.74 | 30.21 | 8 | 0.028 |
| P05156 | Complement factor I | 1.7 | **↓** | 429 | 2 | 59000 | 5.65 | 63487.35 | 7.38 | 20.17 | 4.46 | 0.03 |
|  |  | 1.4 |  | 594 | 3 | 43000 | 4.98 |  |  | 30.16 | 7.72 | 0.023 |
|  |  | 1.5 |  | 453 | 1 | 58000 | 5.89 |  |  | 10.14 | 2.23 | 0.025 |
| P08603 | Complement factor H | 1.2 | **↓** | 57 | 32 | 140000 | 5.47 | 137052.59 | 6.12 | 320.27 | 39.64 | 0.012 |
|  |  | 1.3 |  | 59 | 44 | 139000 | 5.55 |  |  | 450.32 | 49.07 | 0.004 |
|  |  | 1.3 |  | 66 | 52 | 137000 | 5.59 |  |  | 520.34 | 51.99 | 0.003 |
|  |  | 1.2 |  | 72 | 53 | 135000 | 5.63 |  |  | 530.33 | 54.35 | 0.007 |
|  |  | 1.6 |  | 619 | 9 | 40000 | 5.63 |  |  | 90.29 | 12.67 | 0.04 |
|  |  | 1.6 |  | 559 | 3 | 460000 | 4.73 |  |  | 30.27 | 4.71 | 0.044 |
|  |  | 3.8 |  | 456 | 3 | 575000 | 5.04 |  |  | 30.27 | 5.2 | 0.03 |
|  |  | 1.7 |  | 429 | 1 | 59000 | 5.65 |  |  | 10.16 | 1.46 | 0.03 |
|  |  | 1.4 |  | 717 | 2 | 34000 | 4.83 |  |  | 20.23 | 3.09 | 0.012 |
|  |  | 1.5 |  | 453 | 9 | 58000 | 5.89 |  |  | 90.25 | 12.51 | 0.025 |
|  |  | 1.3 |  | 731 | 1 | 34000 | 4.97 |  |  | 10.17 | 1.46 | 0.03 |
|  |  | 1.6 |  | 406 | 1 | 61000 | 6.02 |  |  | 10.16 | 1.5 | 0.028 |
| Q15517 | Corneodesmosin | 1.3 | **↓** | 59 | 1 | 139000 | 5.55 | 48317.16 | 8.48 | 10.13 | 3.4 | 0.004 |
|  |  | 1.6 |  | 619 | 1 | 40000 | 5.63 |  |  | 10.15 | 3.4 | 0.04 |
| P09543 | 2',3'-cyclic-nucleotide 3'-phosphodiesterase | 1.3 | **↓** | 59 | 2 | 139000 | 5.55 | 47578.63 | 9.17 | 20.26 | 5 | 0.004 |
|  |  | 1.2 |  | 57 | 1 | 140000 | 5.47 |  |  | 10.28 | 4.99 | 0.012 |
| P99999 | Cytochrome c | 1.2 | **↓** | 57 | 1 | 140000 | 5.47 | 11617.53 | 9.59 | 10.14 | 12.38 | 0.012 |
| P01040 | Cystatin-A | 1.3 | **↓** | 731 | 1 | 34000 | 4.97 | 10875.3 | 5.39 | 10.16 | 12.24 | 0.03 |
| P81605 | Dermcidin | 1.6 |  | 619 | 1 | 40000 | 5.63 | 9259.29 | 5.64 | 10.13 | 10 | 0.037 |
|  |  | 1.4 | **↓** | 717 | 1 | 34000 | 4.83 |  |  | 10.14 | 10 | 0.012 |
|  |  | 1.4 |  | 581 | 1 | 44000 | 4.8 |  |  | 10.13 | 10 | 0.014 |
| Q02413 | Desmoglein-1 | 1.4 | **↓** | 717 | 4 | 34000 | 4.83 | 107703.49 | 4.77 | 40.23 | 4.67 | 0.012 |
|  |  | 1.2 |  | 72 | 1 | 135000 | 5.63 |  |  | 10.21 | 1.81 | 0.007 |
| P15924 | Desmoplakin | 1.2 | **↓** | 72 | 12 | 135000 | 5.63 | 331773.89 | 6.44 | 70.26 | 4.81 | 0.007 |
|  |  | 1.4 |  | 717 | 10 | 34000 | 4.83 |  |  | 100.23 | 4.56 | 0.012 |
| P14625 | Endoplasmin | 1.2 | **↓** | 57 | 1 | 140000 | 5.47 | 92469 | 4.73 | 10.16 | 2.37 | 0.012 |
| P58107 | Epiplakin | 1.2 | **↓** | 72 | 1 | 135000 | 5.63 | 555620.96 | 5.44 | 10.18 | 1.77 | 0.007 |
| P02671 | Fibrinogen alpha chain | 1.4 | **↓** | 581 | 1 | 44000 | 4.8 | 91358.87 | 5.79 | 10.22 | 2.08 | 0.014 |
| P20930 | Filaggrin | 1.4 | **↓** | 717 | 4 | 34000 | 4.83 | 435169.55 | 9.24 | 40.19 | 4.16 | 0.012 |
| Q5D862 | Filaggrin-2 | 1.4 | **↓** | 717 | 5 | 34000 | 4.83 | 248072.58 | 8.45 | 50.31 | 4.94 | 0.012 |
|  |  | 1.6 |  | 559 | 1 | 46000 | 4.73 |  |  | 10.13 | 0.5 | 0.044 |
|  |  | 1.3 |  | 59 | 1 | 139000 | 5.55 |  |  | 10.33 | 0.79 | 0.004 |
|  |  | 1.5 |  | 453 | 1 | 58000 | 5.89 |  |  | 10.14 | 0.5 | 0.025 |
| P47929 | Galectin-7 | 1.2 | **↓** | 72 | 2 | 135000 | 5.63 | 14943.86 | 7 | 20.18 | 18.38 | 0.007 |
|  |  | 1.4 |  | 717 | 1 | 34000 | 4.83 |  |  | 10.22 | 11.76 | 0.012 |
| P04406 | Glyceraldehyde-3-phosphate dehydrogenase | 1.2 | **↓** | 72 | 2 | 135000 | 5.63 | 35922.02 | 8.58 | 20.15 | 8.36 | 0.007 |
|  |  |  |  |  | 1 | 34000 | 4.97 |  |  | 10.19 | 4.18 | 0.03 |
|  |  |  |  |  | 1 | 34000 | 4.83 |  |  | 10.18 | 4.18 | 0.012 |
| P14136 | Glial fibrillary acidic protein | 1.3 |  | 59 | 1 | 139000 | 5.55 | 49880.21 | 5.42 | 8.18 | 2.55 | 0.004 |
|  |  | 1.3 | **↓** | 66 | 1 | 137000 | 5.59 |  |  | 8.16 | 2.55 | 0.003 |
|  |  | 1.2 |  | 72 | 1 | 135000 | 5.63 |  |  | 8.15 | 2.55 | 0.007 |
|  |  | 1.6 |  | 619 | 1 | 40000 | 5.63 |  |  | 8.17 | 2.55 | 0.04 |
|  |  | 1.4 |  | 717 | 1 | 34000 | 4.83 |  |  | 8.17 | 2.55 | 0.012 |
|  |  | 1.2 |  | 57 | 1 | 140000 | 5.47 |  |  | 8.14 | 2.55 | 0.012 |
|  |  | 1.6 |  | 384 | 1 | 65000 | 6.75 |  |  | 8.18 | 2.55 | 0.03 |
|  |  | 1.6 |  | 376 | 1 | 65000 | 6.65 |  |  | 8.14 | 2.55 | 0.049 |
|  |  | 3.8 |  | 456 | 1 | 58000 | 5.01 |  |  | 8.19 | 2.55 | 0.033 |
| P11021 | Glucose regulated-protein,78kDa | 1.2 | **↓** | 57 | 1 | 140000 | 5.47 | 72333 | 5.01 | 10.13 | 2.75 | 0.012 |
| P22352 | Glutathione peroxidase 3 | 1.7 | **↓** | 429 | 1 | 59000 | 5.65 | 23463.74 | 7.85 | 10.14 | 6.64 | 0.03 |
| P10809 | Heat shock protein,60kDa,mitochondrial | 1.2 | **↓** | 57 | 3 | 140000 | 5.47 | 61055 | 5.24 | 30.28 | 8.2 | 0.012 |
| P02790 | Hemopexin | 1.3 | **↑** | 66 | 2 | 137000 | 5.59 | 49295.43 | 6.43 | 20.26 | 6.49 | 0.003 |
|  |  | 1.4 |  | 594 | 4 | 43000 | 4.98 |  |  | 40.21 | 12.77 | 0.023 |
|  |  | 1.6 |  | 406 | 17 | 61000 | 6.02 |  |  | 170.28 | 54.3 | 0.028 |
|  |  | 1.6 |  | 384 | 10 | 65000 | 6.75 |  |  | 100.26 | 30.52 | 0.03 |
|  |  | 1.6 |  | 376 | 4 | 65000 | 6.65 |  |  | 40.26 | 11.47 | 0.049 |
|  |  | 3.1 |  | 463 | 1 | 57000 | 5.08 |  |  | 10.2 | 3.25 | 0.018 |
|  |  | 3.8 |  | 456 | 2 | 58000 | 5.01 |  |  | 20.17 | 7.79 | 0.033 |
| P04196 | Histidine-rich glycoprotein | 1.4 | **↓** | 594 | 1 | 43000 | 4.98 | 57659.9 | 7.03 | 10.16 | 2.67 | 0.023 |
| Q86YZ3 | Hornerin | 1.2 |  | 72 | 12 | 135000 | 5.63 | 282389.92 | 10.05 | 120.27 | 13.96 | 0.077 |
|  |  | 3.8 |  | 456 | 2 | 57500 | 5.04 |  |  | 20.2 | 4.91 | 0.03 |
|  |  | 1.2 |  | 57 | 1 | 140000 | 5.47 |  |  | 10.14 | 0.42 | 0.012 |
|  |  | 1.3 |  | 66 | 1 | 137000 | 5.59 |  |  | 10.21 | 1.68 | 0.003 |
|  |  | 1.3 |  | 731 | 1 | 34000 | 4.97 |  |  | 10.16 | 0.42 | 0.03 |
|  |  | 1.7 |  | 429 | 1 | 59000 | 5.65 |  |  | 10.17 | 1.68 | 0.03 |
|  |  | 1.4 |  | 594 | 1 | 43000 | 4.98 |  |  | 10.19 | 0.77 | 0.023 |
|  |  | 1.6 |  | 619 | 3 | 40000 | 5.63 |  |  | 30.24 | 2.77 | 0.04 |
| P01871 | Ig mu chain C region | 1.6 | **↑** | 376 | 4 | 65000 | 6.65 | 49307 | 6.34 | 40.17 | 11.95 | 0.049 |
| P19823 | Inter-alpha-trypsin inhibitor heavy chain H2 | 1.3 | **↓** | 59 | 2 | 139000 | 5.55 | 106463 | 5.75 | 20.16 | 3.38 | 0.004 |
|  |  | 1.2 |  | 57 | 1 | 140000 | 5.47 |  |  | 10.13 | 1.29 | 0.012 |
| P14923 | Junction plakoglobin | 1.2 | **↓** | 72 | 1 | 135000 | 5.63 | 81744.74 | 5.75 | 10.14 | 1.61 | 0.007 |
|  |  | 1.4 |  | 717 | 4 | 34000 | 4.83 |  |  | 40.14 | 5.1 | 0.012 |
| P01042 | Kininogen-1 | 3.8 | **↑** | 456 | 1 | 575000 | 5.04 | 69896.7 | 6.23 | 10.14 | 2.64 | 0.029 |
| P02686 | Myelin basic protein | 1.3 | **↓** | 59 | 1 | 139000 | 5.55 | 33117.1 | 9.79 | 10.17 | 3.95 | 0.004 |
|  |  | 1.2 |  | 72 | 1 | 135000 | 5.63 |  |  | 10.18 | 3.95 | 0.007 |
|  |  | 1.5 |  | 453 | 1 | 58000 | 5.89 |  |  | 10.14 | 3.95 | 0.025 |
|  |  | 1.7 |  | 429 | 1 | 59000 | 5.65 |  |  | 10.19 | 3.95 | 0.03 |
|  |  | 1.6 |  | 559 | 1 | 460000 | 4.73 |  |  | 10.21 | 3.95 | 0.044 |
|  |  | 1.6 |  | 406 | 1 | 61000 | 6.02 |  |  | 10.13 | 3.3 | 0.028 |
| P08571 | Monocyte differentiation antigen CD14 | 1.7 | **↓** | 429 | 2 | 59000 | 5.65 | 37214.7 | 5.58 | 20.17 | 7.47 | 0.03 |
|  |  |  |  |  | 4 | 575000 | 5.04 |  |  | 40.28 | 18.13 | 0.03 |
| Q96PD5 | N-acetylmuramoyl-L-alanine amidase | 1.6 | **↑** | 406 | 5 | 61000 | 6.02 | 59980.32 | 7.64 | 50.26 | 15.3 | 0.028 |
|  |  | 3.1 |  | 463 | 3 | 57000 | 5.08 |  |  | 30.27 | 10.76 | 0.018 |
| Q06830 | Peroxiredoxin-1 | 1.2 | **↓** | 72 | 1 | 135000 | 5.63 | 22110.36 | 8.27 | 10.13 | 5.53 | 0.007 |
| P00734 | Prothrombin | 1.7 | **↓** | 429 | 2 | 59000 | 5.65 | 65308.2 | 5.23 | 20.21 | 5.31 | 0.03 |
|  |  | 1.6 |  | 559 | 4 | 460000 | 4.73 |  |  | 40.24 | 10.45 | 0.044 |
|  |  | 1.6 |  | 619 | 1 | 40000 | 5.63 |  |  | 10.13 | 1.93 | 0.04 |
|  |  | 1.4 |  | 581 | 1 | 44000 | 4.8 |  |  | 10.16 | 3.05 | 0.014 |
| P02760 | Protein AMBP | 1.3 | **↓** | 731 | 6 | 34000 | 4.97 | 38999.37 | 6.15 | 60.24 | 17.9 | 0.03 |
|  |  | 1.4 |  | 717 | 6 | 34000 | 4.83 |  |  | 60.24 | 17.9 | 0.012 |
|  |  | 1.2 |  | 57 | 1 | 140000 | 5.47 |  |  | 10.14 | 3.41 | 0.012 |
|  |  | 1.6 |  | 559 | 1 | 46000 | 4.73 |  |  | 10.15 | 3.41 | 0.044 |
| P31151 | Protein S100-A7 | 1.2 | **↓** | 72 | 3 | 135000 | 5.63 | 11332.52 | 6.26 | 30.18 | 30.69 | 0.007 |
| P06702 | Protein S100-A9 | 1.2 | **↓** | 72 | 2 | 135000 | 5.63 | 13110.8 | 5.71 | 20.22 | 24.56 | 0.007 |
|  |  | 1.6 |  | 559 | 1 | 46000 | 4.73 |  |  | 10.22 | 13.16 | 0.044 |
| P02808 | Statherin | 1.6 | **↓** | 559 | 1 | 46000 | 4.73 | 5219.76 | 6.25 | 10.17 | 48.39 | 0.044 |
| P02743 | Serum amyloid P-component | 1.6 | **↓** | 619 | 3 | 40000 | 5.63 | 23258.53 | 6.12 | 30.17 | 15.7 | 0.04 |
|  |  | 1.3 |  | 66 | 1 | 137000 | 5.59 |  |  | 10.14 | 5.83 | 0.003 |
|  |  | 1.7 |  | 429 | 3 | 59000 | 5.65 |  |  | 30.18 | 17.04 | 0.03 |
| P49908 | Selenoprotein P | 1.5 | **↓** | 453 | 2 | 58000 | 5.89 | 41232.26 | 7.95 | 20.15 | 3.41 | 0.025 |
|  |  | 1.7 |  | 429 | 2 | 59000 | 5.65 |  |  | 20.16 | 3.41 | 0.03 |
| P10599 | Thioredoxin | 1.3 | **↓** | 59 | 1 | 139000 | 5.55 | 11606.3 | 4.82 | 10.13 | 12.38 | 0.004 |
|  |  | 1.3 |  | 731 | 1 | 34000 | 4.97 |  |  | 10.16 | 12.38 | 0.03 |
|  |  | 1.4 |  | 717 | 1 | 34000 | 4.83 |  |  | 10.15 | 12.38 | 0.012 |
| P05543 | Thyroxine-binding globulin | 3.8  3.1 | **↑** | 456  463 | 1  1 | 57500  57000 | 5.05  5.08 | 49729.69 | 5.6 | 10.18  10.14 | 3.51  2.68 | 0.03  0.018 |
| Q71U36 | Tubulin alpha-1A chain | 1.2 | **↓** | 57 | 1 | 140000 | 4.94 | 50135.63 | 4.94 | 10.24 | 5.76 | 0.03 |
| P62987 | Ubiquitin-60S ribosomal protein L40 | 1.3 | **↓** | 731 | 1 | 34000 | 4.8 | 14728 | 7 | 10.17 | 12.5 | 0.03 |
|  |  | 1.4 | **↓** | 717 | 1 | 34000 | 4.83 |  |  | 10.18 | 12.5 | 0.012 |
| Q14157 | Ubiquitin-associated protein 2-like | 1.2 | **↓** | 57 | 1 | 140000 | 5.47 | 114534.5 | 6.6 | 10.17 | 1.38 | 0.012 |
| P04070 | Vitamin K-dependent protein C | 1.4 |  | 594 | 3 | 43000 | 4.98 | 47332.75 | 5.6 | 30.26 | 13.23 | 0.023 |
|  |  | 1.4 | **↓** | 581 | 6 | 44000 | 4.8 |  |  | 60.3 | 22.56 | 0.014 |
|  |  | 1.6 |  | 559 | 5 | 46000 | 4.73 |  |  | 50.26 | 20.61 | 0.044 |
| P02774 | Vitamin D-binding protein | 1.7 | **↑** | 429 | 3 | 59000 | 5.65 | 51243.46 | 5.22 | 30.26 | 12.03 | 0.03 |
|  |  | 3.1 |  | 463 | 2 | 57000 | 5.08 |  |  | 20.19 | 8.23 | 0.018 |
|  |  | 1.7 |  | 804 | 4 | 30000 | 6.04 |  |  | 40.24 | 12.66 | 0.001 |
| P12955 | Xaa-Pro dipeptidase | 1.5 | **↓** | 453 | 7 | 58000 | 5.89 | 54416.98 | 5.64 | 70.23 | 18.05 | 0.025 |
| P25311 | Zinc-alpha-2-glycoprotein | 1.3 | **↓** | 66 | 4 | 137000 | 5.59 | 32144.94 | 5.58 | 40.19 | 18.1 | 0.003 |
|  |  | 1.7 |  | 429 | 4 | 59000 | 5.65 |  |  | 40.17 | 15.1 | 0.03 |
|  |  | 1.4 |  | 594 | 14 | 43000 | 4.98 |  |  | 140.25 | 42.62 | 0.023 |
|  |  | 1.4 |  | 581 | 13 | 44000 | 4.8 |  |  | 130.32 | 42.28 | 0.014 |
|  |  | 1.6 |  | 559 | 8 | 46000 | 4.73 |  |  | 80.31 | 30.2 | 0.044 |
|  |  | 1.6 |  | 406 | 6 | 61000 | 6.02 |  |  | 60.2 | 25.5 | 0.028 |
|  |  | 1.6 |  | 619 | 1 | 40000 | 5.63 |  |  | 10.19 | 4.03 | 0.04 |

^a^Refers to the fold change in spot volume between responders and non responders observed in the 2D-DIGE experiment

^b^ Refers to the master spot number on the 2D-DIGE gel assigned in Progenesis

^c,d^ MW and pI values derived from Swiss-Prot Compute pI/MW tool (<http://www.expasy.org/tools/pi_tool.html>) ( MW reported in Daltons)

^e^ Amino acid sequence coverage
